# Theoretical Investigation of Single-Molecule-Magnet Behavior in Mononuclear Dysprosium and Californium Complexes

**DOI:** 10.1021/acs.inorgchem.2c04013

**Published:** 2023-01-18

**Authors:** Debmalya Ray, Meagan S. Oakley, Arup Sarkar, Xiaojing Bai, Laura Gagliardi

**Affiliations:** † Department of Chemistry, Chemical Theory Center, and Minnesota Supercomputing Institute, University of Minnesota, Minneapolis, Minnesota55455, United States; ‡ Department of Chemistry, Pritzker School of Molecular Engineering, James Franck Institute, Chicago Center for Theoretical Chemistry, The University of Chicago, Chicago, Illinois60637, United States

## Abstract

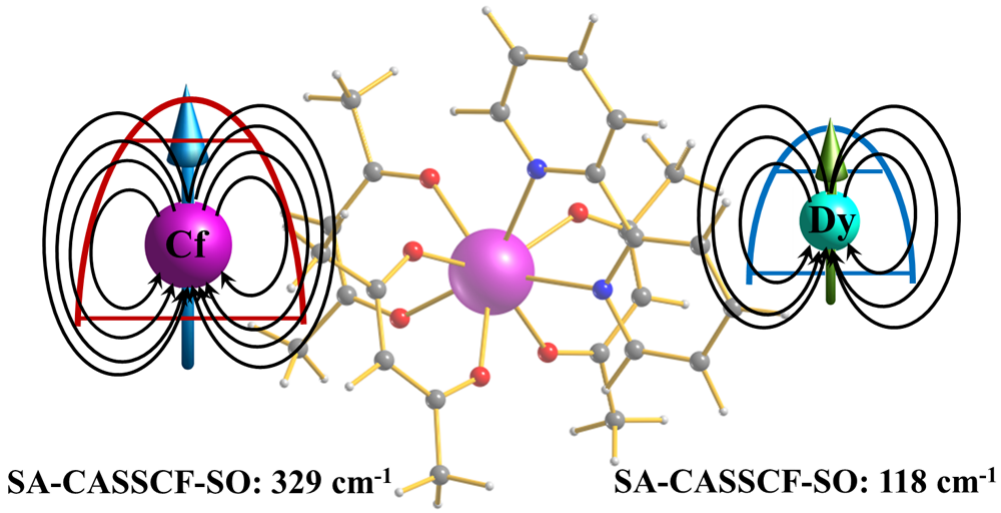

Early-actinide-based (U, Np, and Pu) single-molecule magnets (SMMs) have yet to show magnetic properties similar to those of highly anisotropic lanthanide-based ones. However, there are not many studies exploring the late-actinides (more than half-filled f shells) as potential candidates for SMM applications. We computationally explored the electronic structure and magnetic properties of a hypothetical Cf(III) complex isostructural to the experimentally synthesized Dy(dbm)_3_(bpy) complex (bpy = 2,2′-bipyridine; dbm = dibenzoylmethanoate) via multireference methods and compared them to those of the Dy(III) analogue. This study shows that the Cf(III) complex can behave as a SMM and has a greater magnetic susceptibility compared to other experimentally and computationally studied early-actinide-based (U, Np, and Pu) magnetic complexes. However, Cf spontaneously undergoes α-decay and converts to Cm. Thus, we also explored the isostructural Cm(III)-based complex. The computed magnetic susceptibility and **g**-tensor values show that the Cm(III) complex has poor SMM behavior in comparison to both the Dy(III) and Cf(III) complexes, suggesting that the performance of Cf(III)-based magnets may be affected by α-decay and can explain the poor performance of experimentally studied Cf(III)-based molecular magnets in the literature. Further, this study suggests that the ligand field is dominant in Cf(III), which helps to increase the magnetization blocking barrier by nearly 3 times that of its 4f congener.

## Abstract 2

Using multireference ab initio calculations, we explored whether the Cf(III)-based complex shows single-molecule-magnet behavior superior to that of the isostructural Dy(III) analogue.

## Introduction

Single-molecule magnets (SMMs) exhibit magnetic hysteresis, a process in which a system becomes magnetized through exposure to a magnetic field and slowly demagnetizes upon removal of the field.^1^ SMMs can become highly magnetized in one of two equilibrium states depending on the direction of the applied magnetic field. The effective magnetic relaxation energy barrier, *U*_eff_, which separates these two bistable magnetic states, scales with the square of the total spin, *S*, and the size of anisotropy, *D*.^2^ Early SMMs were composed of polynuclear transition-metal clusters to maximize *S*, but magnetic hysteresis was observed at only very low temperatures (4 K).^3−5^

In the case of transition-metal complexes, ligand-field effects dominate the splitting of the ground and excited states; hence, the spin–orbit coupling is small, and the nature of the magnetic bistability can be defined by spin substates, *m*_*s*_.^6^ For lanthanides, the spin–orbit coupling dominates over the ligand field,^7^ and the states are composed of *m*_*J*_ sublevels, which is the projection of the total angular momentum, *J*, along the magnetic anisotropy axis. The energy gap between the ground and first excited *m*_*J*_ states can be increased further through crystal-field (CF) splitting, and thus *U*_eff_ may also increase.^8−11^ Both the large magnetic moments and unquenched orbital angular momentum of lanthanides are crucial properties in designing SMMs with higher blocking temperatures (*T*_B_) closer to room temperature. Dysprosium metallocenes have been at the forefront of lanthanide SMM research,^12−15^ with large *U*_eff_ barriers (up to 1541 cm^–1^) and magnetic blocking temperatures reported above the liquid-nitrogen temperature (*T*_B_ = 80 K).^16^

Extensive research has been performed to understand how to engineer lanthanide-based SMMs with ideal magnetic properties,^17−20^ but fewer studies have been performed on actinides.^21−24^ Because actinides have much larger spin–orbit coupling constant values than lanthanides, actinide-based SMMs can potentially produce greater magnetic anisotropy barriers and magnetic moments upon the systematic design of ligands.^25^ Additionally, the greater radial extent of the 5f orbitals compared to that of the 4f orbitals^21,26−28^ increases the likelihood of covalency between the actinide and ligand (and therefore partial quenching of the angular momentum), which can produce strong magnetic exchange.^29,30^ These unique features require new design techniques to be developed specifically for late-actinide-based SMMs.

The most common actinide-based SMMs contain uranium (due to its abundance and stability), but they have yet to reach the success of highly anisotropic lanthanide-based SMMs.^9,30−39^ There are much greater challenges associated with synthesizing and characterizing SMMs containing actinides rather than lanthanides because they are less accessible, expensive, and hazardous to handle. However, computational chemistry provides a safe alternative to experimental actinide chemistry and the opportunity to determine and understand design criteria for actinide-based SMMs, allowing this field to grow more rapidly.

Complexes containing 5f-block metals are generally multireference and have large spin–orbit coupling, so it is not surprising that there are serious limitations of density functional theory (DFT) in computing ground- or excited-state properties of uranium complexes.^40^ One way to approximately account for these characteristics is to use the complete active space self-consistent field (CASSCF) with spin–orbit coupling (CASSCF-SO); CASSCF-SO has been shown previously to be successful in predicting magnetic susceptibilities of actinide-based SMMs.^23,33,41−43^ Recently, Goodwin et al.^44^ isolated and characterized a californium metallocene complex, which opens up the possibility of Cf(III) to act as a potential candidate for SMM applications. The magnetic properties of a few Cf(III) compounds have been measured,^45−47^ and some computational studies of the electronic structure of Cf(III) complexes have recently been published,^44,45,48,49^ but, to the best of our knowledge, there are no computational studies of the magnetic properties of Cf(III) SMMs.

In this work, we determined the magnetic properties of a Cf(III) complex which is isostructural to the previously synthesized Dy(dbm)_3_(bpy) complex (bpy = 2,2′-bipyridine; dbm = dibenzoylmethanoate).^50,51^ There are few reports of Cf(III) complexes in the literature, and further Cf(III)-based SMMs are also not reported. Here we chose a simplified model of the Dy(III) complex and the Cf(III) analogue to make the calculations affordable. Because Cf(III) can easily undergo α-decay and convert to Cm(III),^45^ we also investigated the isostructural Cm(III) complex to determine the effect of this ligand field on different trivalent actinides and how it affects the performance of Cf(III)-based magnets. Therefore, this study on isoelectronic Dy(III) and Cf(III) complexes could open up possibilities to study other Cf(III)-based SMMs both computationally and experimentally in the near future.

## Computational Methods

### DFT Calculations

**Figure 1:**
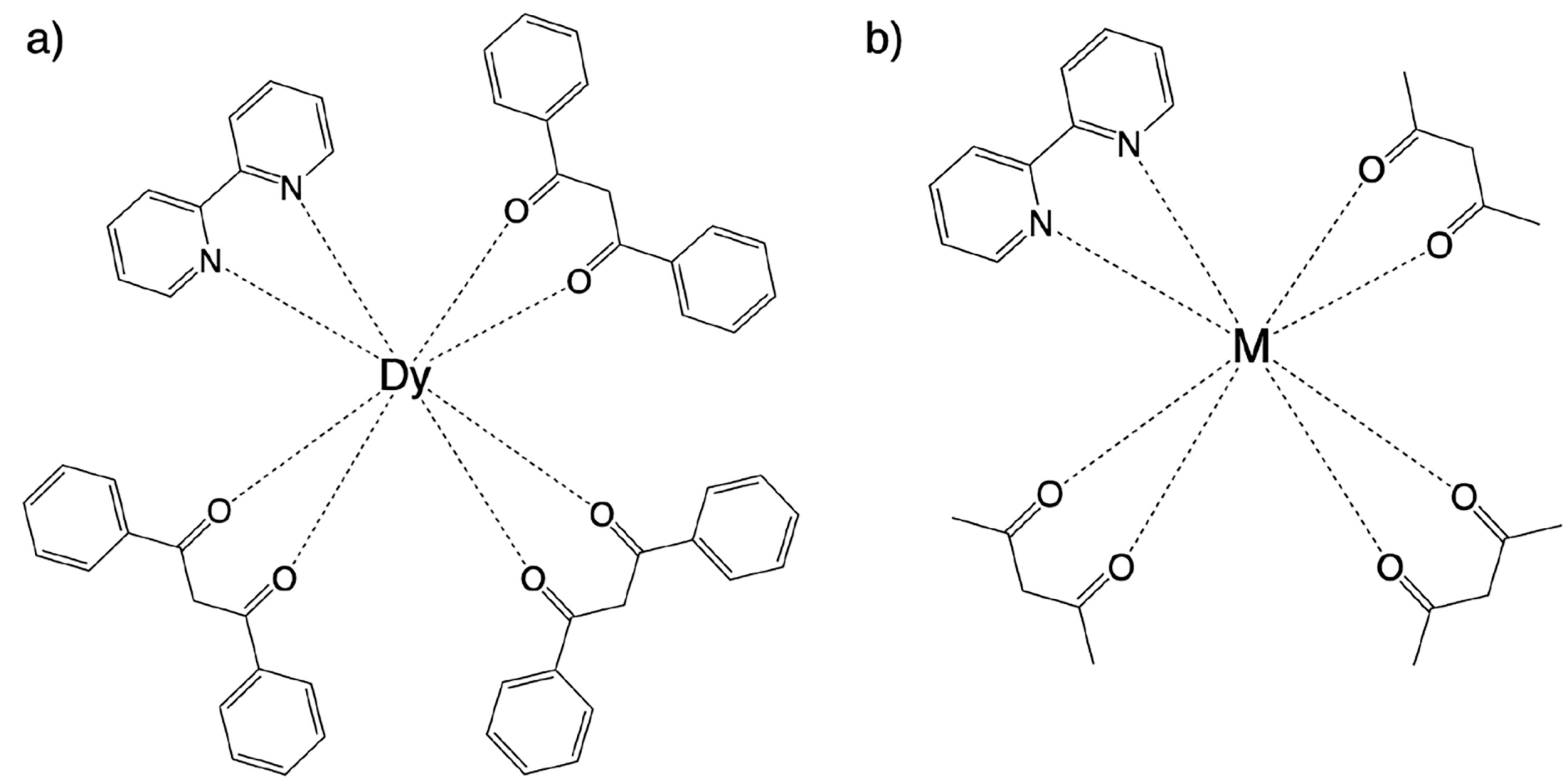
Schematic representations of the (a) Dy-Ph (**1**_ph_) and (b) M-Me [M = Dy^III^ (**1**_me_), Cf^III^ (**2**_me_), and Cm^III^ (**3**_me_)] complexes.

The experimental crystal structure of the Dy(dbm)_3_(bpy) complex^51^ (referred to here as Dy-Ph; Figure 1a) was used as an initial structure for all of the DFT geometry optimizations. In order to reduce the computational cost, the phenyl rings of the dibenzoylmethanoate linkers in the Dy-Ph complex were replaced with methyl groups. We will refer to this truncated complex as Dy-Me (Figure 1b). The Dy(III) ion was replaced with Cf(III) and Cm(III) in the optimized truncated complex to generate the Cf-Me and Cm-Me structures. Geometry optimizations of the highest spin state (sextet for Dy and Cf and octet for Cm) for the Dy-Ph, Dy-Me, Cf-Me, and Cm-Me complexes were performed with DFT using the BP86 functional,^52^ which has been shown to predict accurate geometries for actinide complexes.^41,42^ The TZ2P basis set was used for the metal centers (Dy, Cf, and Cm) and the DZP basis set for the C, H, O, and N atoms.^53^ The zero-order regular approximation (ZORA) was used to include scalar relativistic effects.^54−56^ All DFT computations were performed using the *ADF2016* software package.^57−59^

### Multireference Calculations

The electronic structures of the Dy-Ph, Dy-Me, Cf-Me, and Cm-Me complexes (at the DFT-optimized geometry) were analyzed using the CASSCF method^60,61^ as implemented in the *OpenMolcas* (version 19.11, tag 1312-g91e1abe) software package.^62^ The resolution of identity Cholesky decomposition^63^ was used to compute the two-electron integrals at a reduced cost. Second-order Douglas–Kroll–Hess (DKH) Hamiltonian was employed to incorporate scalar relativistic effects, together with relativistic all-electron basis sets. Two different basis set approaches were used. The first consisted of the cc-pVDZ-DK3 basis set for the metal centers (Dy, Cf, and Cm)^64,65^ and the cc-pVDZ-DK basis set for the H, C, N, and O atoms^66,67^ (referred to here as BS1). The second basis set consisted of the cc-pVTZ-DK3 basis sets for the metal centers (Dy, Cf, and Cm),^64,65^ the cc-pVTZ-DK basis set for the N and O atoms^66,67^ and the cc-pVDZ-DK basis sets for the C and H atoms (referred to here as BS2).

All metals are in the formal 3+ oxidation state, and Dy(III), Cf(III), and Cm(III) have the valence electronic configurations 4f^9^, 5f^9^, and 5f^7^, respectively. We performed state-averaged CASSCF (SA-CASSCF) calculations with an active space that includes all f electrons and f orbitals. This results in a (9,7) active space for the Dy and Cf complexes and a (7,7) active space for the Cm complex. For the Dy and Cf complexes, the (9,7) active space gives rise to 21 sextet, 224 quartet, and 490 doublet states, which were all included in the SA-CASSCF calculations within their respective spin symmetry. For the Cm complex, the (7,7) active space generates one octet, 48 sextet, 392 quartet, and 784 doublet states. All of the octet, sextet, and quartet configurations and the first 600 doublet states are included in the SA-CASSCF calculations within their respective spin symmetry. Moreover, for the Cf(III) complex, we also performed SA-CASSCF calculations by including the five 6d orbitals for a CAS(9,12) active space using 21 sextets and 128 quartets only.

State interaction was described via the restricted-active-space self-interaction (RASSI) method.^68^ For the Dy and Cf complexes, 21 sextet, 128 quartet, and 130 doublet states were included in the RASSI calculation, and for the Cm complex, 1 octet, 21 sextet, 119 quartet, and 41 doublet states were included in the RASSI calculation. These states were included based on a selected energy cutoff, where there was a large energy gap between the highest excited state included and the next excited state. An effective one-electron Fock-type spin–orbit Hamiltonian was used. Two-electron terms were treated as screening corrections of the one-electron terms. The atomic-mean-field integrals, as implemented in *OpenMolcas*, were employed.^69^ The spin–orbit interaction was computed *a posteriori* to SA-CASSCF (SA-CASSCF-SO).

The effect of dynamic correlation was included using extended multistate complete active space second-order perturbation (XMS-CASPT2) theory.^70−72^ Recent work on Dy(III) complexes by Reta et al.^73^ showed that when only 21 sextet roots (and no other spin states) from the SA-CASSCF calculation (referred to here as SA-CASSCF-low) are coupled with RASSI (SA-CASSCF-SO-low), they give similar results in terms of the magnetic properties compared to similar calculations using 21 sextet, 128 quartet, and 130 doublet roots. Thus, in order to reduce the computational cost at the XMS-CASPT2 level, we use the above protocol and compute only 21 sextet roots for the Dy-Me and Cf-Me complexes. The NOMULT keyword in *OpenMolcas* was used to reduce the computational cost by disallowing state mixing. Three groups of CASSCF sextet states, 11, 7, and 3 states, which correspond to the , , and  terms, respectively, were used to run three independent XMS-CASPT2 computations. This was done to retain a degeneracy that is artificially lifted by introducing mixing between the states and state-averaging with multistate and extended multistate approaches. Spin–orbit coupling was then accounted for with RASSI (XMS-CASPT2-SO). This approach was previously used in the multireference study of other Dy(III) compounds.^16^

The *SINGLE_ANISO* program^74−76^ was employed to compute **g** tensors, magnetic blocking barriers, magnetic susceptibility (χ*T*) curves using the van Vleck formalism,^77^ and magnetic moments (μ) of the spin–orbit-coupled states. The CF Hamiltonian that is projected on the eight ground-state Kramers doublets (KDs) of 2*J* + 1 eigenfunctions is expressed as^6,78^
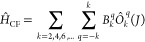
1where *Ô*_*k*_^*q*^ are the extended Stevens operators^79^ and *B*_*k*_^*q*^ are the CF parameters of rank *k* = 2, 4, and 6.^6^ The *B*_2_^0^, *B*_4_^0^, and *B*_6_^0^ parameters indicate the axial CF splitting, which helps to increase the axial anisotropy of the system, while the nonaxial terms *B*_2_^±1±2^, *B*_4_^±1,±2,±3,±4^, and *B*_6_^±1,±2,±3,±4,±5,±6^ denote the transverse aniostropy in the complex. The nonzero CF terms are determined by the symmetry or point group of the ion in question, particularly the first coordination sphere around the metal center.^6^ The blocking barrier diagrams are plotted in the paper with respect to the relative energies of the KDs, which connect (via the magnetic moment operator) the intra-KD and inter-KD states with the QTM, TA-QTM, and Orbach/Raman probabilities. The absolute values of the transition probabilities or the transition magnetic dipole moments were computed using the *SINGLE_ANISO* module^80^ according to the expression
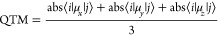
2where μ_*x*_, μ_*y*_, and μ_*z*_ are the components of the total magnetic moment, μ, and *i* and *j* are spin–orbit-coupled KD states, where *i* ≠ *j*.

## Results and Discussion

### Structural Analysis of the Dy-Ph, Dy-Me, Cf-Me, and Cm-Me Complexes

To determine the accuracy of our predicted structures, we first compared the DFT-computed Dy–N and Dy–O bond lengths of the Dy-Ph complex to the experimental values (X-ray structure), as reported in Table 1. Here the experimental crystal structure is denoted as Dy-Ph (or **1**_ph_), and the DFT-optimized geometry is denoted as Dy-Ph(DFT) (or **1**_ph_^opt^). The computed bond lengths are within 0.02 Å of the experimental values. This suggests that the BP86 functional gives reasonable bond distances, and this protocol was used for the truncated model complexes Dy-Me (or **1**_me_), Cf-Me (or **2**_me_), and Cm-Me (or **3**_me_). The replacement of the phenyl ring with the methyl group does not change significantly the Dy–N and Dy–O bond lengths. The Cf–N/O and Cm–N/O bond lengths are slightly elongated (less than 0.1 Å difference) compared with the corresponding Dy ones (Table 1).

**Table 1:** M–N (Å) and M–O (Å) Bond Lengths in the **1**_ph_, **1**_ph_^opt^, 1_me_, **2**_me_, and **3**_me_ Complexes

complex	M–N (Å)	M–O (Å)
**1**_ph_	2.576	2.314
**1**_ph_^opt^	2.599	2.323
**1**_me_	2.604	2.327
**2**_me_	2.636	2.368
**3**_me_	2.672	2.394

### Magnetic Properties of **1**_ph_ and **1**_ph_^opt^ Complexes

We first discuss complexes **1**_ph_ and **1**_ph_^opt^ shown in Figure 1. The ground-state electronic configuration of the Dy(III) free ion has a term symbol . For the **1**_ph_ complex, from the SA-CASSCF calculations, the sextet state is the ground state and the quartet and doublet states lie 24966 and 37470 cm^–1^ above the sextet ground state, respectively (Figure S1). The sextet, quartet, and doublet spin states span energy ranges of 0–35327, 24966–107293, and 37470–180563 cm^–1^, respectively. There is a 12081 cm^–1^ energy gap between the 128th and 129th quartet spin states and a 2749 cm^–1^ gap between the 130th and 131st doublet spin states. Thus, in the RASSI calculation, we included the first 21 sextet, 128 quartet, and 130 doublet states (overall covering a ∼50000 cm^–1^ energy window). At the **1**_ph_^opt^ geometry, the energy differences before inclusion of spin–orbit coupling are similar to those at the **1**_ph_ geometry. The SA-CASSCF-SO relative energies of the ground and excited spin states of complexes **1**_ph_ and **1**_ph_^opt^ are shown in Table 2 (also in Table S1).

**Table 2:** Relative Energies (cm^–1^) of the Lowest Nine Spin–orbit States, KDs, of **1**_ph_ and **1**_ph_^opt^ Using SA-CASSCF-SO and the BS2 Basis Sets

KD state	**1**_ph_	**1**_ph_^opt^
KD1	0.0	0.0
KD2	159.7	117.3
KD3	220.5	155.7
KD4	251.4	197.6
KD5	299.4	235.6
KD6	341.8	288.8
KD7	407.6	380.1
KD8	493.4	496.1
KD9	3636.7	3590.1

**Figure 2:**
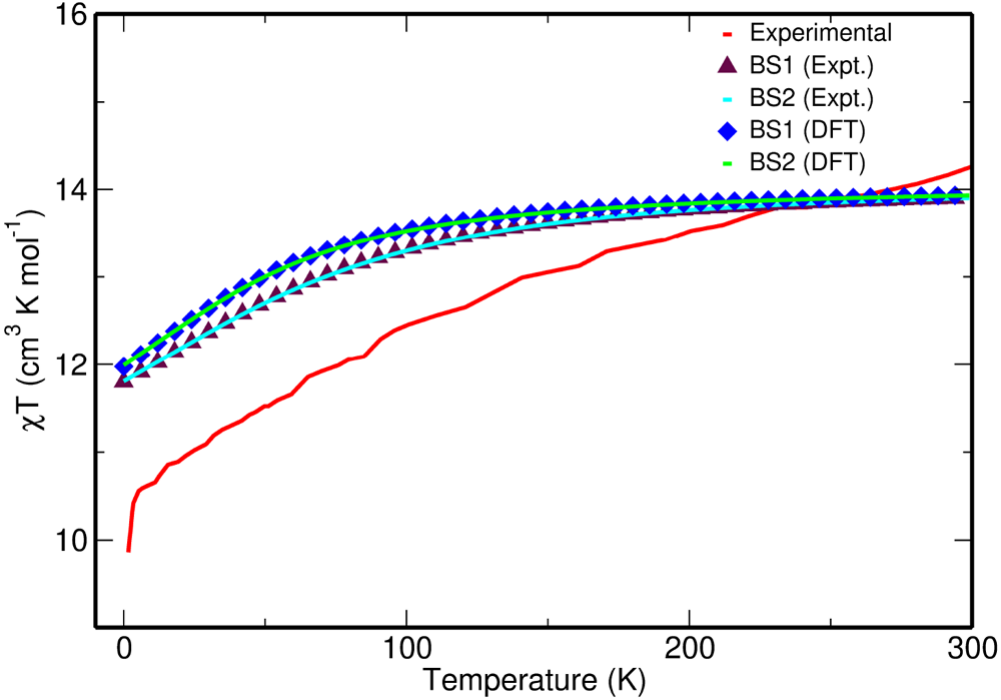
Comparison of the experimental and computed χ*T* curves as a function of *T* for both complexes **1**_ph_ and **1**_ph_^opt^, computed at the SA-CASSCF-SO level with the BS1 and BS2 basis sets.

We then computed the magnetic susceptibility curve for complexes **1**_ph_ and **1**_ph_^opt^, and in both cases, the value at 0 K is overestimated compared to the experiment (Figure 2). The discrepancy between the theoretically computed χ*T* and the experimental value may be due to the fact that neither full dynamic correlation in the electronic structure calculation nor intermolecular exchange interactions within the unit cell in the magnetic susceptibility simulations are incorporated.

**Figure 3:**
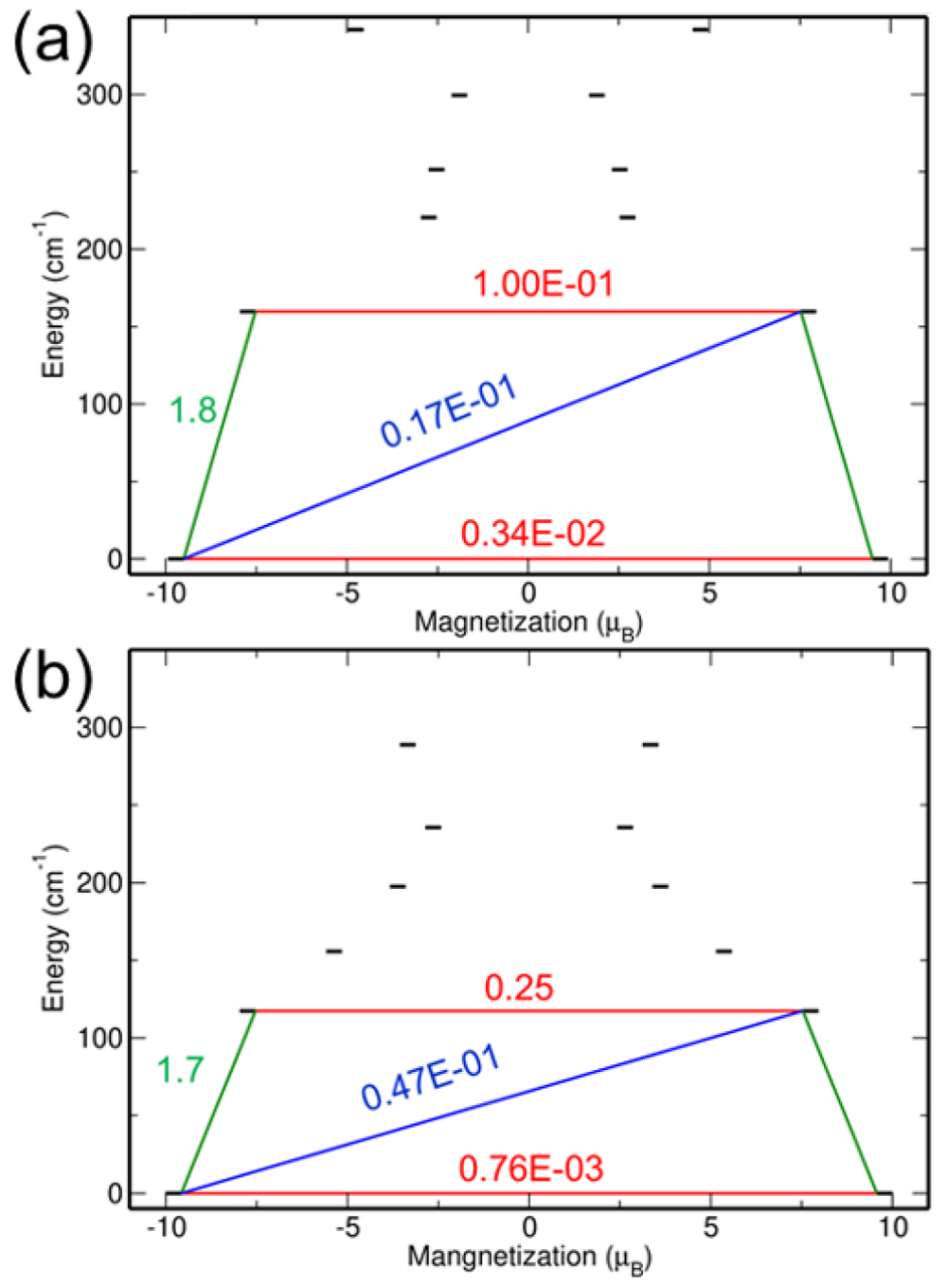
Comparison of the blocking barriers of (a) **1**_ph_ and (b) **1**_ph_^opt^ using SA-CASSCF-SO with the BS2 basis set. The red lines indicate quantum tunneling of magnetization (QTM) or termally assisted QTM (TA-QTM) processes between |±*m*_*J*_⟩ states. The green and blue lines indicate the transitions between the inter-KDs (via Orbach and/or Raman mechanisms). The values correspond to transition magnetic moment matrix elements (in μ_B_) between the *m*_*J*_ levels.

Using BS2, the computed blocking barrier height is 159.7 cm^–1^ for **1**_ph_ and 117.3 cm^–1^ for **1**_ph_^opt^. The blocking barrier plots for both complexes are shown in Figure 3. These plots are generated by computing the transition magnetic moment matrix elements in the basis of the *m*_*J*_ multiplets using the *SINGLE_ANISO* code. The *g* values for the ground-state KDs show highly uniaxial anisotropy, which is one of the necessary criteria for good SMM behavior. The *g* values for the first eight KDs using BS2 (and BS1) are reported in Table 3 (and in Table S3). The calculations performed with either BS1 and BS2 predict similar magnetic properties (Figure 2 and Table S2). Thus, only the BS2 results are discussed in the main paper, and the BS1 results are presented in the Supporting Information.

**Table 3:** Comparison of *g* Values for **1**_ph_ and **1**_ph_^opt^ Complexes at the SA-CASSCF-SO Level with the BS2 Basis Set

	**1**_ph_			**1**_ph_^opt^		
KD state	*g*_*x*_	*g*_*y*_	*g*_*z*_	*g*_*x*_	*g*_*y*_	*g*_*z*_
KD1	0.00	0.01	19.43	0.00	0.00	19.58
KD2	0.23	0.36	15.63	0.62	0.80	16.84
KD3	2.46	3.40	13.72	0.97	1.78	13.52
KD4	8.93	5.81	1.33	3.47	4.94	8.11
KD5	2.08	3.72	12.97	2.69	4.21	9.88
KD6	0.84	1.30	17.47	0.12	0.32	17.39
KD7	0.09	0.28	18.58	0.07	0.13	18.43
KD8	0.02	0.06	19.39	0.01	0.02	19.48

In order to understand the various competing magnetic relaxation processes, we analyzed the transition magnetic moments between the intra-KD (between the ±*m*_*J*_ levels) and inter-KDs (between the *m*_*J*_ and *m*_*J*–1_ levels). The intra-KD transition or the expectation value of ⟨+*m*_*J*_|μ|−*m*_*J*_⟩ is known as QTM, and for the excited-state KDs, the intra-KD transition is called thermally assisted QTM or TA-QTM. The largest transition magnetic moment matrix element connecting the KDs indicates the most probable pathway of magnetic relaxation. In the case of complex **1**_ph_^opt^, the ground state is |±^15^/_2_⟩ and the transverse magnetic moment between |+^15^/_2_⟩ to |−^15^/_2_⟩ is on the order of 10^–3^ μ_B_ (Figure 3). The transition magnetic moments are higher between the |±^15^/_2_⟩ and |±^13^/_2_⟩ states compared to that of the QTM between the |+^15^/_2_⟩ and |−^15^/_2_⟩ states, which suggests that at higher temperatures excited *m*_*J*_ state(s) will be accessible and magnetic relaxation may take place via TA-QTM. Because the TA-QTM at the first excited state is significant and greater or equal to 0.1 μ_B_, the magnetization in both the **1**_ph_^opt^ and **1**_ph_ complexes is likely to relax via the first excited-state KD.

### Effect of Linker Truncation

**Figure 4:**
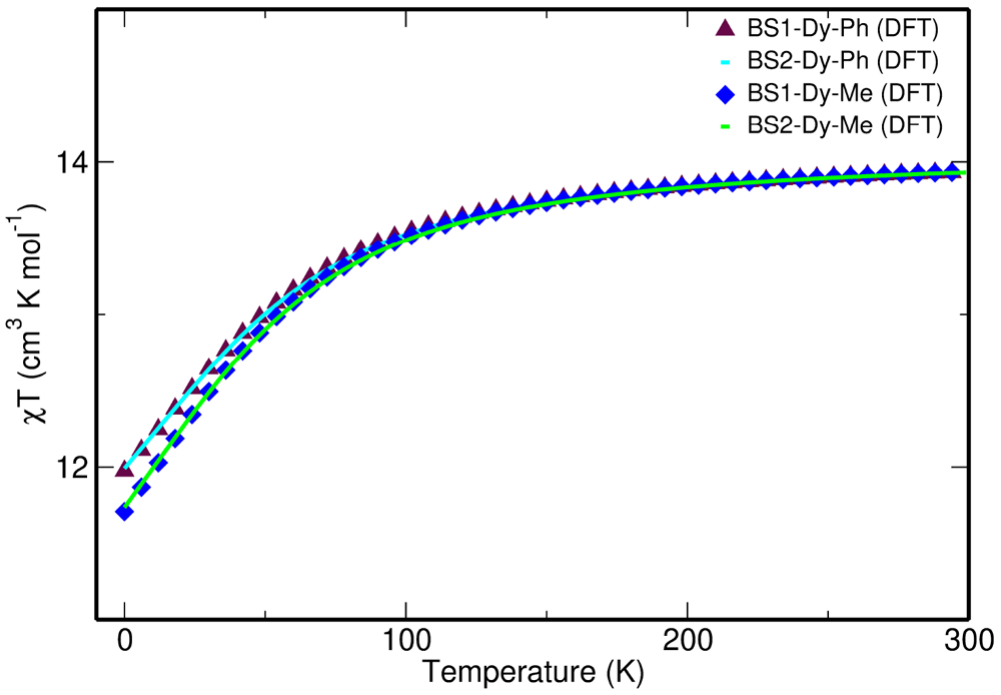
Comparison of the χ*T* curves of complexes **1**_ph_^opt^ [or Dy-Ph(DFT)] and **1**_me_ (or Dy-Me) using the SA-CASSCF-SO method with the BS1 and BS2 basis sets.

In order to reduce the computational cost, the phenyl linkers of dibenzoylmethanoate were truncated to methyl groups. As shown in Table 1, truncation of the ligands corresponds to a negligible change in the bond lengths in the first coordination sphere. We further investigated the effect of linker truncation on the magnetic properties of the Dy(III) complexes. As shown in Figure 4, linker truncation barely affects the magnetic susceptibility curves at the BS1 and BS2 SA-CASSCF-SO levels of theory. The energies of the first nine KDs and **g**-tensor values for both the **1**_ph_^opt^ and **1**_me_ complexes are reported in Tables S3–S5. These tables show that linker truncation does not affect the magnetic properties of these Dy(III) magnets, and hence this truncation scheme can serve as a good model for exploring the magnetic properties of complexes containing other metals such as Cf(III) and Cm(III) while maintaining computational efficiency.

### Comparison of the Magnetic Properties of **1**_me_, **2**_me_, and **3**_me_

At the SA-CASSCF level of theory, in the energy spectrum of the **1**_me_ complex, the sextet, quartet, and doublet states spanned over 0–35315, 24953–107279, and 37439–180547 cm^–1^, respectively, which is similar to that of the **1**_ph_ complex. For the **2**_me_ complex, the SA-CASSCF energy windows for the sextet, quartet, and doublet spin states are 0–25981, 18857–78804, and 28562–132354 cm^–1^, respectively (Figure S2). For **1**_me_ and **2**_me_, there are gaps of 12906 and 7907 cm^–1^, respectively, between the 128th and 129th quartet spin states and gaps of 2805 and 985 cm^–1^, respectively, between the 130th and 131st doublet spin states. Similar to the **1**_ph_ complex, we also included 21 sextet, 128 quartet, and 130 doublet states in the RASSI-SO calculations for the other complexes. The energies of the lowest nine KDs are reported in Table 4. The CF splitting between the ground state and the first excited state is ∼200 cm^–1^ larger in **2**_me_ than in **1**_me_. This is expected because actinides exert a stronger crystal field than lanthanides due to the larger radial extension of the 5f orbitals. As in the **1**_ph_ case, there is a large gap in energy between the eighth and ninth KDs for both the **1**_me_ and **2**_me_ complexes (Table 4). Thus, we included only the first eight KDs when computing the anisotropic barrier of the **2**_me_ complex.

**Table 4:** Relative Energies (cm^–1^) of the Lowest Nine KDs of **1**_me_, **2**_me_, and **3**_me_ Using the SA-CASSCF-SO Method with the BS2 Basis Set

	**1**_me_	**2**_me_	**3**_me_
KD1	0.0	0.0	0.0
KD2	118.3	329.0	5.8
KD3	169.6	398.9	9.4
KD4	199.9	481.0	13.2
KD5	232.0	544.8	26141.2
KD6	278.3	664.2	26296.3
KD7	356.7	813.7	26411.7
KD8	490.8	1107.7	26681
KD9	3599.4	8280.9	28414.6

**Figure 5:**
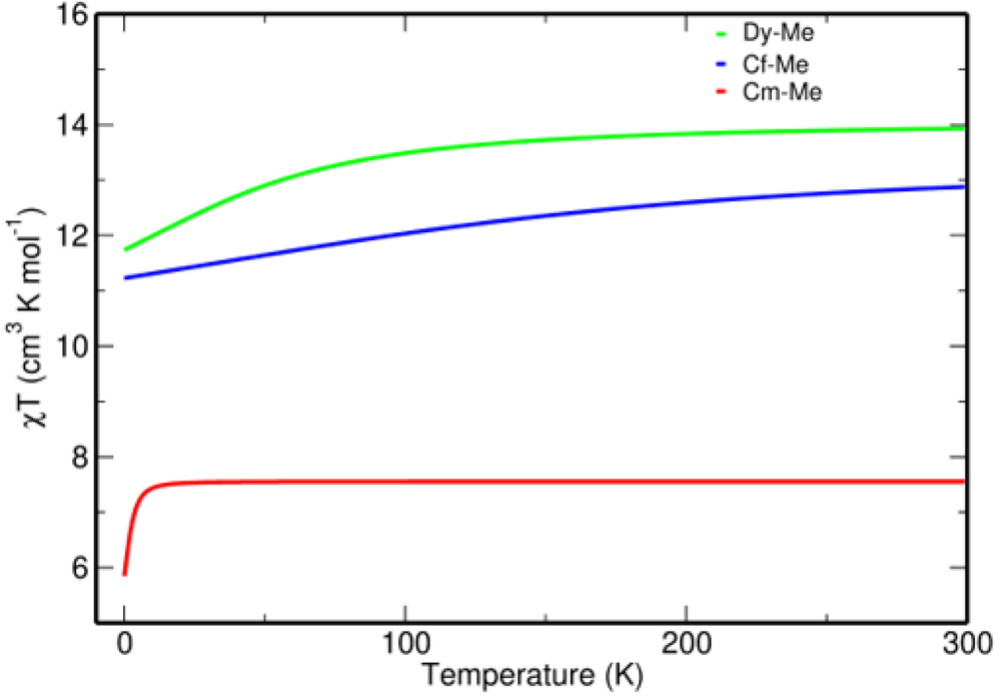
Comparison of the computed χ*T* versus *T* curves of the **1**_me_ (or Dy-Me), **2**_me_ (or Cf-Me), and **3**_me_ (or Cm-Me) complexes using the SA-CASSCF-SO method with the BS2 basis set.

The magnetic susceptibility curves for **1**_me_ and **2**_me_ are shown in Figure 5. The χ*T* value for **2**_me_ is slightly lower than that of the **1**_me_ complex over the 0–300 K temperature range. This can be attributed to the larger CF splitting of Cf(III) compared to the Dy(III) species, which causes a reduction in the χ*T* value. Also, in Table 4, it is seen that the energy separations between the *m*_*J*_ states are higher in the case of Cf-Me compared to Dy-Me, which suggests a steeper decrease in the χ*T* curve in accordance with subsequent depopulation of the *m*_*J*_ states at lower temperatures. A similar difference has been previously observed between Cf_2_O_3_ and Dy_2_O_3_.^47^ Moreover, the magnetic susceptibility of the free Cf(III) ion is 9.7 cm^3^ K mol^–1^, whereas that of Dy(III) is 10.2 cm^3^ K ^–1^ at approximately 0 K.^47^ The χ*T* value of the Cf-Me complex at 300 K is at least 10 times higher than those of other early-actinide-based SMMs.^23,41,42^ This is because Cf(III) has a ^6^H_15/2_ ground state [similar to Dy(III)], which has the largest *g* factor in combination with the highest *J* value.^81^ The relative energies of the first few KDs (Table 4) indicate that the blocking barrier of the **1**_me_ and **2**_me_ complexes are around 118.3 and 329.0 cm^–1^, respectively. The **g**-tensor values corresponding to the ground-state KD of the **1**_me_ complex are *g*_*x*_ = *g*_*y*_ = 0.01 and *g*_*z*_ = 19.37, similar to those of the **2**_me_ complex, *g*_*x*_ = *g*_*y*_ = 0.0 and *g*_*z*_ = 18.95 (Table 5). Both **1**_me_ and **2**_me_ exhibit highly axial magnetic anisotropy (Tables 5 and S6). The *g*_*z*_ axis for the ground-state KD for both **1**_me_ and **2**_me_ point toward the same direction (Figure S3). This suggests that **2**_me_ has a magnetic behavior similar to that of **1**_me_, and **2**_me_ may behave as a suitable SMM candidate. The *g*_*z*_ angle of the first excited-state KD is ∼18° in both complexes, indicating possible relaxation via the first excited-state KD (Table 5).

**Table 5:** Comparison of *g* Values, *g*_*z*_ Angles of the Ground State and First Excited-State KD Energies and Wavefunction Decomposition of **1**_me_ and **2**_me_ from the SA-CASSCF-SO Method with the BS2 Basis Set

complex	energy of the KDs (cm^–1^)	*g*_*x*_	*g*_*y*_	*g*_*z*_	*g*_*z*_ angle (deg)	wavefunction {*m*_*J*_}
**1**_me_	0.0	0.011	0.012	19.376		91%|±^15^/_2_⟩
	118.3	0.428	0.534	15.931	18.0	68%|±^13^/_2_⟩, 16%|±^9^/_2_⟩
**2**_me_	0.0	0.007	0.009	18.951		93%|±^15^/_2_⟩
	329.0	0.858	1.410	14.538	18.2	64%|±^13^/_2_⟩, 19%|±^9^/_2_⟩

**Figure 6:**
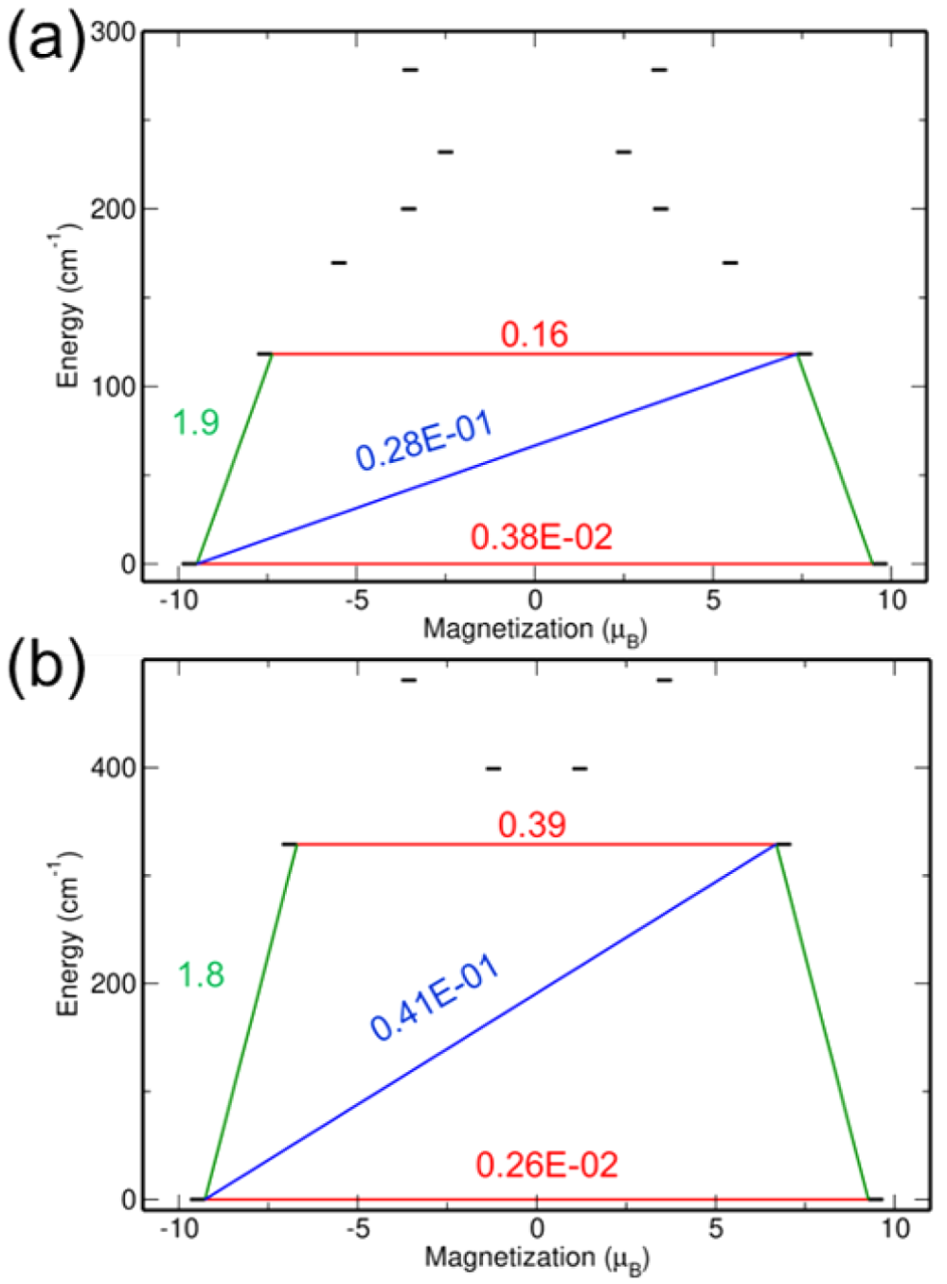
Comparison of the blocking barriers for (a) **1**_me_ and (b) **2**_me_ computed using SA-CASSCF-SO and the BS2 basis set. The red lines indicate QTM or TA-QTM processes between |±*m*_*J*_⟩ states. The green and blue lines indicate the transitions between the inter-KDs (via Orbach and/or Raman mechanisms). The values correspond to transition magnetic moment matrix elements (μ_B_) between the *m*_*J*_ levels.

The blocking barrier is reported for both the **1**_me_ and **2**_me_ complexes in Figure 6. In both cases, the transition magnetic moments from the |±^15^/_2_⟩ to |±^13^/_2_⟩ states (shown in green in Figure 6) are higher than the ground-state QTM process. For the **1**_me_ and **2**_me_ complexes, the magnetic relaxation will likely take place via the first excited state through the TA-QTM process (Figure 6). Further, the magnetic blocking barrier of **2**_me_ is 211 cm^–1^ higher than that of **1**_me_, suggesting that the magnetic relaxation may be slower in the case of the **2**_me_ complex. It is important to mention that the methods used here to compute the barrier to magnetic reversal do not account for the spin–lattice relaxation processes explicitly. The *SINGLE_ANISO* module computes only the mixing coefficients between the intra- and inter-KDs, and thus the transition magnetic moments shown in Figures 3 and 6 only account for the static picture of the magnetic relaxation.

To further rationalize the enhancement in the computed blocking barrier height of **2**_me_ compared to **1**_me_, the ab initio CF parameters obtained from the *SINGLE_ANISO* module were analyzed.^76^ We also investigated the effect of the basis set on the magnetic susceptibility, relative energy of KDs, and **g**-tensor and blocking barrier values (Figures S4–S6 and Tables S7 and S8). Both basis sets (BS1 and BS2) used in this work give similar values. From Table S9, it is clearly seen that **2**_me_ has larger contributions from the axial CF parameters (*B*_2_^0^, *B*_4_^0^, and *B*_6_^0^) compared to the 4f congener, which supports the fact that the **2**_me_ complex has a stronger axial anisotropy arising from stronger CF splitting. Additionally, the nonaxial or transverse CF parameters are high in both complexes, which indicates significant mixing of the components of the ground-state *J* = ^15^/_2_ manifold (Table 5). Possibly due to this reason, the ground-state QTM for both complexes are small but nonnegligible, and this causes the higher excited-state TA-QTM values to be high and allows relaxation from the first excited-state KD.

In order to understand the effect of the 6d orbitals on the spin–orbit states, we have performed a CAS(9,12) calculation for the **2**_me_ complex. The results show that, upon the incorporation of the five virtual 6d orbitals into the active space, the spin–orbit energy states are higher in energy compared to the CAS(9,7) active space results (Table S10). For instance, the energy of the first excited-state KD increases by 100 cm^–1^. This behavior is also observed in previous cases in the Pu(III) system^42^ and is not unexpected because the empty 6d orbitals were separated by a large energy gap (0.4 hartree in the DFT level) from the 5f orbitals in the **2**_me_ complex. This is a typical situation that occurs in active space-based calculations, when one cannot use a complete active space. Perhaps the definite way to do it would be to perform CASPT2 on top of the different active spaces, and one would probably see converged results. However, CASPT2 calculations with so many states are not feasible. To summarize, we think that the inclusion of 5f → 6d excitations may deteriorate the quality of blocking barrier calculations for the Cf(III) complex at the CASSCF level, compared with the calculations including only the 5f orbitals in the active space.

Cf(III) readily undergoes α-decay and converts to Cm(III);^45^ thus, we also explored the magnetic properties of the **3**_me_ complex. Our study shows that, for Cm(III), the octet spin state is very stable and the *J* = ^7^/_2_ state is the ground state with the term symbol . The computed magnetic susceptibility (Figure 5) of the **3**_me_ complex is significantly lower and flatter than that of the **2**_me_ complex, and the *g* values are also less anisotropic (Table S6). Moreover, the first four KDs are extremely close in energy (within 13 cm^–1^). This is because the orbital angular momentum is zero for Cm(III) at the ground state, and the sextet excited states lie more than 26000 cm^–1^ away from the octet ground state (Table 4). This suggests that the magnetic properties of the **2**_me_ complex will be lost if Cf(III) decays to Cm(III).

### Effect of Dynamic Correlation on the Magnetic Properties of the **1**_me_ and **2**_me_ Complexes

Similar to Reta et al.,^73^ we first compared the magnetic properties of the Dy-Me and Cf-Me complexes using the SA-CASSCF-SO (including 21 sextet, 128 quartet, and 130 doublet states) and SA-CASSCF-SO-low (including only the lowest 21 sextet states) levels of theory. Our results show a negligible change in the magnetic susceptibility (Figure S6) and energies of the lowest eight KDs (Table S11) for **1**_me_ but a larger shift in the magnetic susceptibility in the case of **2**_me_. Furthermore, without the quartet and doublet roots, the ninth KD energy for **2**_me_ is underestimated by 2500 cm^–1^. However, the energy of the ninth KD is still higher by ∼3000 cm^–1^ (for **1**_me_) and ∼5700 cm^–1^ (for **2**_me_) at the SA-CASSCF-SO-low level, and hence we decided not to include it in the magnetic property calculation.

Next, we compared the energy spectrum of the 21 sextet roots using XMS-CASPT2 to that of SA-CASSCF. Although it would be desirable to include the lower spin states in the XMS-CASPT2 calculations, this is unaffordable due to the huge computational cost. Our results show that the energy window of the sextet decreases by ∼7000 and ∼6400 cm^–1^ for the **1**_me_ and **2**_me_ complexes, respectively, at the XMS-CASPT2 level compared to SA-CASSCF (Table S12).

**Figure 7:**
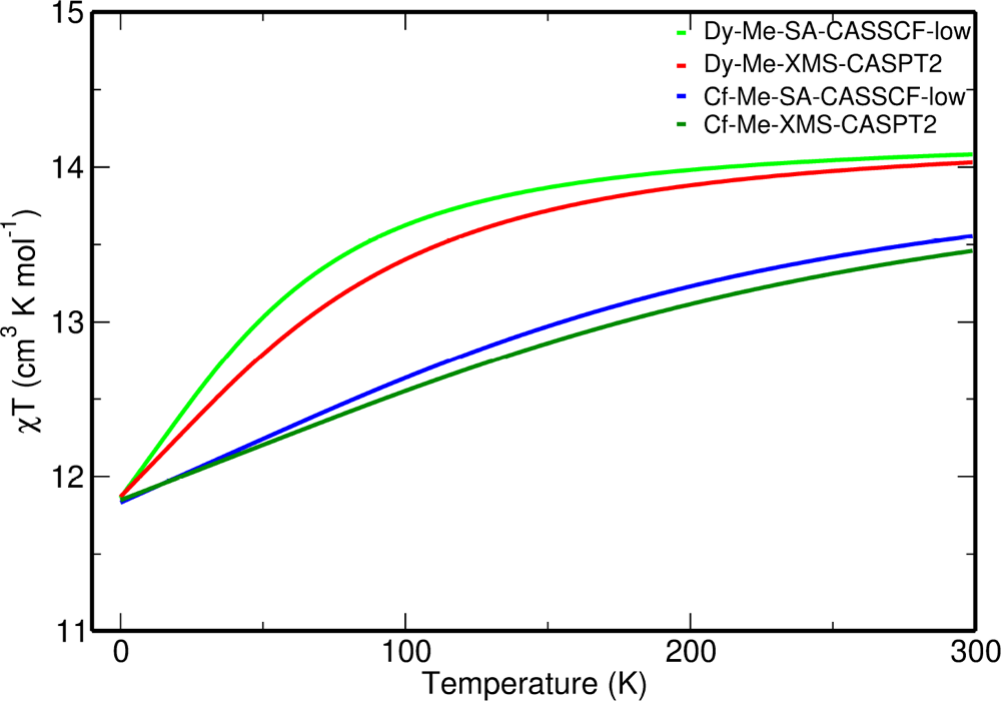
Comparison of the computed χ*T* versus *T* curves of the **1**_me_ (or Dy-Me) and **1**_me_ (or Cf-Me) complexes using the SA-CASSCF-SO-low and XMS-CASPT2-SO methods and the BS2 basis set.

The XMS-CASPT2-SO magnetic susceptibility curve is similar to the SA-CASSCF-SO-low one (Figure 7). We also note that the energies of the first eight KDs are similar at the two levels of theory (Table 6). At all levels of theory, SA-CASSCF-SO, SA-CASSCF-SO-low, and XMS-CASPT2-SO, **1**_me_ and **2**_me_ undergo magnetic relaxation via the first excited-state KDs. The XMS-CASPT2-SO-computed barrier heights are 162.0 and 418.6 cm^–1^ for **1**_me_ and **2**_me_, respectively, and 120.1 and 363.1 cm^–1^ using SA-CASSCF-SO-low. A further comparison of the **g**-tensor values in Table S13 also shows that both the **1**_me_ and **2**_me_ complexes are highly anisotropic at the XMS-CASPT2-SO level of theory.

**Table 6:** Relative Energies (cm^–1^) of the First Nine KDs of **1**_me_ and **2**_me_ Using the SA-CASSCF-SO-low and XMS-CASPT2-SO Levels of Theory (Using the BS2 Basis Set)

	**1**_me_		**2**_me_	
	SA-CASSCF-SO-low	XMS-CASPT2-SO	SA-CASSCF-SO-low	XMS-CASPT2-SO
KD1	0.0	0.0	0.0	0.0
KD2	120.1	162.0	363.1	418.6
KD3	171.3	232.2	406.3	473.2
KD4	201.9	265.4	516.6	599.4
KD5	233.7	304.3	581.5	675.6
KD6	283.1	374.5	741.3	854.8
KD7	363.0	464.3	911.2	1049.7
KD8	499.2	622.7	1238.6	1404.3
KD9	3045.4	3076.5	5864.6	5904.4

## Conclusion

We explored the electronic and magnetic properties of a not yet synthesized Cf(III) complex, isostructural to the experimentally synthesized Dy(dbm)_3_(bpy) complex (bpy = 2,2′-bipyridine; dbm = dibenzoylmethanoate) via multireference methods and compared the two systems. Both the Dy(III) and Cf(III) species show promising SMM properties, namely, highly uniaxial magnetic anisotropy and magnetic bistability. Due to the inherently stronger spin–orbit coupling and CF splitting present in actinide-based complexes, the computed blocking barrier height of the Cf(III) species is higher than that of the Dy(III) analogue. Analysis of the *g* values and electronic structures shows similar behavior of the two species. The axial CF parameters and relative energies of the KDs point toward stronger CF splitting in the Cf(III) species, which can have a major influence on the magnetic relaxation behavior. By α-decay, the Cf(III) complex would spontaneously convert into the Cm(III) analogue, which, according to our calculations, would not retain the favorable magnetic properties of Cf(III). This is the first study of a hypothetical Cf(III) complex able to mimic the behavior of Dy-based SMMs. We believe that this study will trigger more experimental work in the field of late-actinide-based SMMs.

## Supporting Information Available

Relevant tables and figures of computed energy plots, **g**-tensor directions, etc. (PDF)
