# Supplementary material for: Theoretical Investigation of Single-Molecule-Magnet Behavior in Mononuclear Dysprosium and Californium Complexes
Source: Inorg Chem. 2023 Jan 18;62(4):1649–58. doi: 10.1021/acs.inorgchem.2c04013 (PMC9890484; doi:10.1021/acs.inorgchem.2c04013)
Supplement: Supplementary file 1 — ic2c04013_si_001.pdf [file ic2c04013_si_001.pdf]

Supporting Information

Theoretical Investigation of Single-Molecule Magnet Behavior in Mononuclear Dysprosium and Californium Complexes

Debmalya Ray,[†] Meagan S. Oakley,[†] Arup Sarkar,[‡] Xiaojing Bai,[†,¶] and Laura Gagliardi[*,‡]

†*Department of Chemistry, Chemical Theory Center, and Minnesota Supercomputing Institute, University of Minnesota, Minneapolis, MN, 55455, United States*

‡*Department of Chemistry, Pritzker School of Molecular Engineering, James Franck Institute, Chicago Center for Theoretical Chemistry, The University of Chicago, Chicago, IL, 60637, United States*

¶*School of Materials Science and Engineering, Anyang Institute of Technology, Anyang, Henan, 455000, China*

E-mail: lgagliardi@uchicago.edu

List of Figures

S1 Relative energies (cm^{-1}) of various roots of $\mathbf{1}_{ph}$ complex computed using SA-CASSCF method. Basis set choice of BS2 was used for these calculations. The first sextet root is taken as the ground state. S4

S2 Relative energies (cm^{-1}) of all the roots of the (a) $\mathbf{1}_{me}$ and (b) $\mathbf{2}_{me}$ complexes as computed using SA-CASSCF level. The BS2 basis set was used for these calculations. The first sextet root is taken as the ground state. S7

S3 The major anisotropic axis, i.e., the g_{zz} axes of the ground state KD of the two complexes- $\mathbf{1}_{me}$ (left) and $\mathbf{2}_{me}$ (right) which point towards the similar direction. This tells us that the direction of magnetic anisotropy exerted by the ligand arrangement are the same in two complexes. Color code: Dy: cyan, Cf: magenta, O: red, N: blue, C: dark grey, H: light grey. The Dy(III) and Cf(III) free ions have oblate f-electron density in the m$_J$=15/2 state. In order to reduce charge contact between the f-electron density and ligand, a stronger donating ligand would prefer an axial position, whereas a weaker donating ligand would prefer an equatorial position. Here, the nitrogen atoms from the bipyridine group, a weaker field ligand, occupy the perpendicular or equatorial position. Thus, the g$_{zz}$ axis is located in the direction as one of the acetate ligands, as oxygen is a stronger field ligand than nitrogen. S8

S4 Comparison of computed χT vs T curve using DFT optimized geometry of $\mathbf{1}_{me}$ (or Dy-Me) and $\mathbf{2}_{me}$ (or Cf-Me) at SA-CASSCF-SO level of theory and BS1 basis set combinations. S9

S5 Comparison of blocking barrier of (a) $\mathbf{1}_{me}$ and (b) $\mathbf{2}_{me}$ computed using SA-CASSCF-SO level of theory and BS1 basis set combinations using SINGLE-ANISO module. The red lines indicate QTM or TA-QTM processes between $|\pm m_J\rangle$ states. The green and the blue lines indicate the transitions between the inter KDs (via Orbach and/or Raman mechanisms). The values correspond to transition magnetic moment matrix elements (in μ_B) between the m_J levels. S10

S6 Comparison of the computed χT vs T curves of $\mathbf{1}_{me}$ (or Dy-Me) and $\mathbf{2}_{me}$ (or Cf-Me) complexes using SA-CASSCF-SO and SA-CASSCF-SO-low level of theory and the BS2 basis set. S12

List of Tables

S1 Relative energies (cm^{-1}) of the first 9 Kramers doublets of $\mathbf{1}_{ph}$ and $\mathbf{1}_{ph}^{opt}$ using SA-CASSCF-SO method with BS1 basis set. S5

S2 Comparison of g-tensor values for $\mathbf{1}_{ph}$ and $\mathbf{1}_{ph}^{opt}$ at the SA-CASSCF-SO level of theory using BS1 set of basis sets. S5

S3 Relative energies (cm^{-1}) of the first 9 Kramers doublets of $\mathbf{1}_{ph}^{opt}$ and $\mathbf{1}_{me}$ using SA-CASSCF-SO level of theory using BS1 and BS2 set of basis sets. S6

S4 Comparison of g-tensor values for $\mathbf{1}_{ph}^{opt}$ and $\mathbf{1}_{me}$ at the SA-CASSCF-SO level of theory using BS1 set of basis sets. S6

S5 Comparison of g-tensor values for $\mathbf{1}_{ph}^{opt}$ and $\mathbf{1}_{me}$ at the SA-CASSCF-SO level of theory using BS2 set of basis sets. S6

S6 Comparison of g-tensor values for $\mathbf{1}_{me}$, $\mathbf{2}_{me}$ and $\mathbf{3}_{me}$ computed using SA-CASSCF-SO level with BS2 basis set. S9

S7 Relative energies (cm^{-1}) of the first 9 Kramers doublets of $\mathbf{1}_{me}$ and $\mathbf{2}_{me}$ using SA-CASSCF-SO level of theory using BS1 set of basis sets. S9

S8 Comparison of g-tensor values for $\mathbf{1}_{me}$ and $\mathbf{2}_{me}$ at the SA-CASSCF-SO level of theory using BS1 set of basis sets. S10

S9 Comparison between the ab initio derived crystal-field parameters for the $\mathbf{1}_{me}$ complex and $\mathbf{2}_{me}$ complex computed at the SA-CASSCF-SO level with BS2 basis set. S11

S10 Comparison between the spin-orbit energies (cm^{-1}) for $\mathbf{2}_{me}$ complex using different active spaces at the SA-CASSCF-SO level with BS2 basis set. . . . S11

S11 Relative energies (cm-1) of the first 9 Kramers doublets of $\mathbf{1}_{me}$ and $\mathbf{2}_{me}$ using SA-CASSCF-SO and SA-CASSCF-SO-low level of theory (using BS2 basis set). S12
S12 Comparison of relative energy (cm-1) of 21 sextet roots of $\mathbf{1}_{me}$ and $\mathbf{2}_{me}$ complex using SA-CASSCF and XMS-CASPT2 level of theory (using BS2 basis set). S13
S13 Computed g-tensor values of $\mathbf{1}_{me}$ and $\mathbf{2}_{me}$ complex using SA-CASSCF-SO-low and XMS-CASPT2-SO level of theory (using BS2 basis set). S13

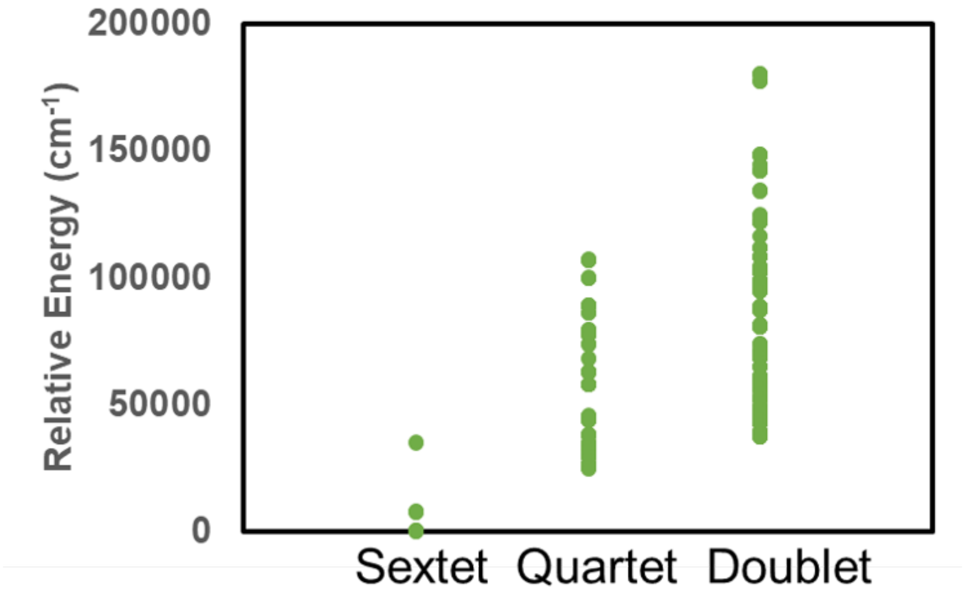

Figure S1: Relative energies (cm-1) of various roots of $\mathbf{1}_{ph}$ complex computed using SA-CASSCF method. Basis set choice of BS2 was used for these calculations. The first sextet root is taken as the ground state.

Table S1: Relative energies (cm^{-1}) of the first 9 Kramers doublets of $\mathbf{1}_{ph}$ and $\mathbf{1}_{ph}^{opt}$ using SA-CASSCF-SO method with BS1 basis set.

	$\mathbf{1}_{ph}$	$\mathbf{1}_{ph}^{opt}$
KD1	0.0	0.0
KD2	156.7	113.9
KD3	214.4	151.2
KD4	243.0	192.5
KD5	289.1	228.0
KD6	339.7	284.9
KD7	392.2	366.4
KD8	469.5	476.0
KD9	3641.9	3595.2

Table S2: Comparison of g-tensor values for $\mathbf{1}_{ph}$ and $\mathbf{1}_{ph}^{opt}$ at the SA-CASSCF-SO level of theory using BS1 set of basis sets.

	$\mathbf{1}_{ph}$			$\mathbf{1}_{ph}^{opt}$		
	g_x	g_y	g_z	g_x	g_y	g_z
KD1	0.01	0.01	19.42	0.00	0.00	19.57
KD2	0.28	0.40	15.58	0.63	0.83	16.85
KD3	3.09	4.30	13.39	1.03	1.81	13.38
KD4	9.32	5.23	0.36	3.43	4.84	8.10
KD5	2.16	3.26	13.61	2.55	4.45	10.02
KD6	0.59	0.89	17.71	0.01	0.19	17.49
KD7	0.13	0.43	18.40	0.08	0.15	18.41
KD8	0.03	0.07	19.38	0.01	0.02	19.48

Table S3: Relative energies (cm-1) of the first 9 Kramers doublets of $\mathbf{1}_{ph}^{opt}$ and $\mathbf{1}_{me}$ using SA-CASSCF-SO level of theory using BS1 and BS2 set of basis sets.

	BS1		BS2	
	$\mathbf{1}_{ph}^{opt}$	$\mathbf{1}_{me}$	$\mathbf{1}_{ph}^{opt}$	$\mathbf{1}_{me}$
KD1	0.0	0.0	0.0	0.0
KD2	113.9	114.3	117.3	118.3
KD3	151.2	164.0	155.7	169.6
KD4	192.5	194.5	197.6	199.9
KD5	228.0	224.7	235.6	232.0
KD6	284.9	271.7	288.8	278.3
KD7	366.4	343.3	380.1	356.7
KD8	476.0	475.8	496.1	490.8
KD9	3595.2	3603.7	3590.1	3599.4

Table S4: Comparison of g-tensor values for $\mathbf{1}_{ph}^{opt}$ and $\mathbf{1}_{me}$ at the SA-CASSCF-SO level of theory using BS1 set of basis sets.

	$\mathbf{1}_{ph}^{opt}$			$\mathbf{1}_{me}$		
	g_x	g_y	g_z	g_x	g_y	g_z
KD1	0	0	19.57	0.01	0.01	19.35
KD2	0.63	0.83	16.85	0.42	0.52	15.93
KD3	1.03	1.81	13.38	1.43	1.85	14.24
KD4	3.43	4.84	8.1	1.39	3.91	8.88
KD5	2.55	4.45	10.02	3.14	5.81	9.75
KD6	0.01	0.19	17.49	0.21	0.4	18.39
KD7	0.08	0.15	18.41	0.03	0.06	19.03
KD8	0.01	0.02	19.48	0	0.01	19.69

Table S5: Comparison of g-tensor values for $\mathbf{1}_{ph}^{opt}$ and $\mathbf{1}_{me}$ at the SA-CASSCF-SO level of theory using BS2 set of basis sets.

	$\mathbf{1}_{ph}^{opt}$			$\mathbf{1}_{me}$		
	g_x	g_y	g_z	g_x	g_y	g_z
KD1	0	0	19.58	0.01	0.01	19.37
KD2	0.62	0.8	16.84	0.43	0.53	15.93
KD3	0.97	1.78	13.52	1.35	1.8	14.25
KD4	3.47	4.94	8.11	1.79	4.22	8.78
KD5	2.69	4.21	9.88	3.07	5.25	9.99
KD6	0.12	0.32	17.39	0.24	0.45	18.34
KD7	0.07	0.13	18.43	0.02	0.05	19.02
KD8	0.01	0.02	19.48	0	0	19.68

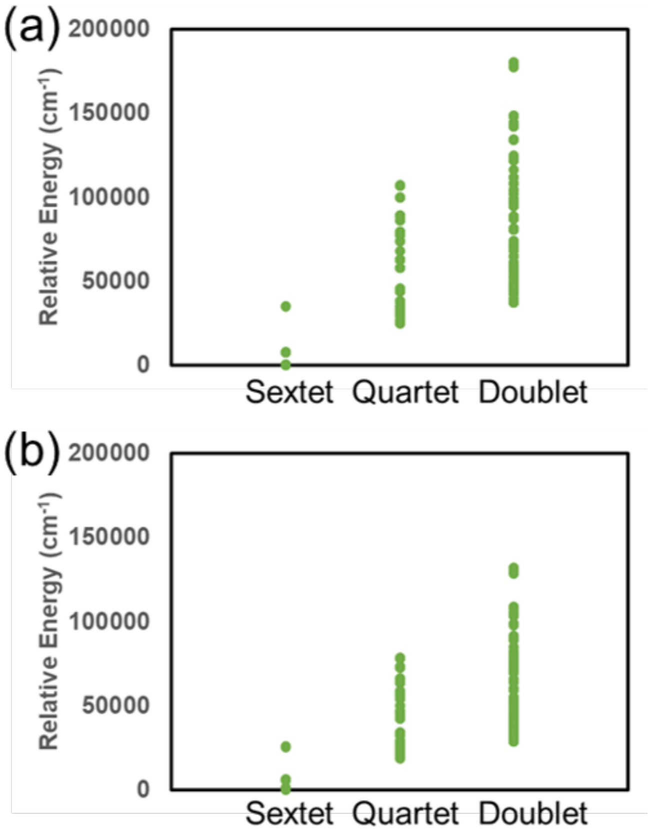

Figure S2: Relative energies (cm^{-1}) of all the roots of the (a) $\mathbf{1}_{me}$ and (b) $\mathbf{2}_{me}$ complexes as computed using SA-CASSCF level. The BS2 basis set was used for these calculations. The first sextet root is taken as the ground state.

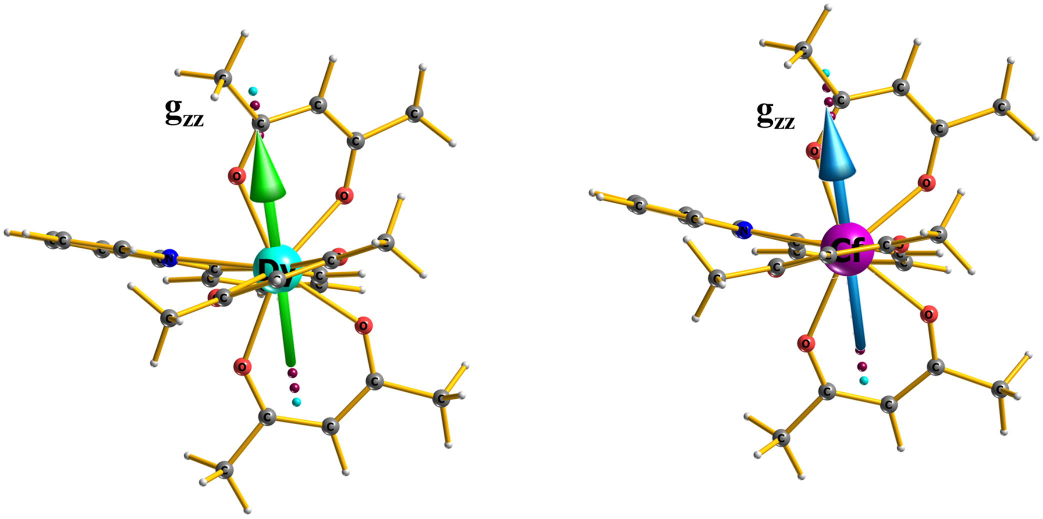

Figure S3: The major anisotropic axis, i.e., the g_{zz} axes of the ground state KD of the two complexes- $\mathbf{1}_{me}$ (left) and $\mathbf{2}_{me}$ (right) which point towards the similar direction. This tells us that the direction of magnetic anisotropy exerted by the ligand arrangement are the same in two complexes. Color code: Dy: cyan, Cf: magenta, O: red, N: blue, C: dark grey, H: light grey. The Dy(III) and Cf(III) free ions have oblate f-electron density in the m_J=15/2 state. In order to reduce charge contact between the f-electron density and ligand, a stronger donating ligand would prefer an axial position, whereas a weaker donating ligand would prefer an equatorial position. Here, the nitrogen atoms from the bipyridine group, a weaker field ligand, occupy the perpendicular or equatorial position. Thus, the g_{zz} axis is located in the direction as one of the acetate ligands, as oxygen is a stronger field ligand than nitrogen.

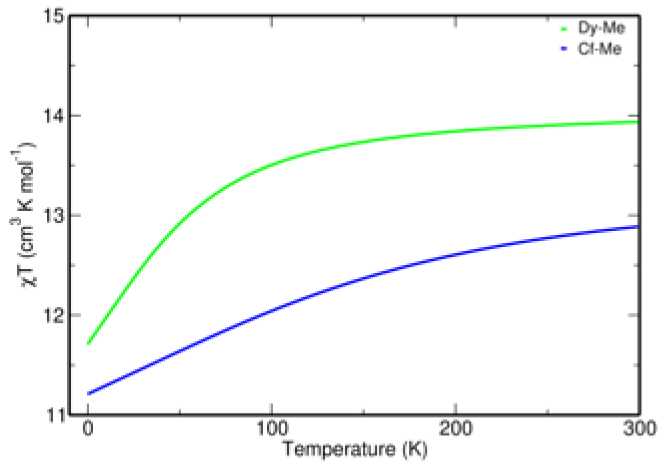

Figure S4: Comparison of computed χT vs T curve using DFT optimized geometry of $\mathbf{1}_{me}$ (or Dy-Me) and $\mathbf{2}_{me}$ (or Cf-Me) at SA-CASSCF-SO level of theory and BS1 basis set combinations.

Table S6: Comparison of g-tensor values for $\mathbf{1}_{me}$, $\mathbf{2}_{me}$ and $\mathbf{3}_{me}$ computed using SA-CASSCF-SO level with BS2 basis set.

	$\mathbf{1}_{me}$			$\mathbf{2}_{me}$			$\mathbf{3}_{me}$		
	g_x	g_y	g_z	g_x	g_y	g_z	g_x	g_y	g_z
KD1	0.01	0.01	19.37	0.00	0.00	18.95	0.15	0.17	13.67
KD2	0.43	0.53	15.93	0.86	1.41	14.54	1.47	1.78	9.51
KD3	1.35	1.80	14.25	1.13	2.13	15.22	3.94	4.69	6.74
KD4	1.79	4.22	8.78	1.21	4.89	8.97	0.63	1.21	12.78
KD5	3.07	5.25	9.99	3.25	4.18	10.01	0.04	1.27	8.57
KD6	0.24	0.45	18.34	0.27	0.44	17.72	0.78	2.94	7.77
KD7	0.02	0.05	19.02	0.03	0.04	18.28	1.22	2.72	6.21
KD8	0.00	0.00	19.68	0.01	0.02	19.04	0.07	1.08	7.98

Table S7: Relative energies (cm^{-1}) of the first 9 Kramers doublets of $\mathbf{1}_{me}$ and $\mathbf{2}_{me}$ using SA-CASSCF-SO level of theory using BS1 set of basis sets.

	$\mathbf{1}_{me}$	$\mathbf{2}_{me}$
KD1	0.0	0.0
KD2	114.3	319.8
KD3	164.0	394.5
KD4	194.5	470.8
KD5	224.7	531.6
KD6	271.7	655.6
KD7	343.3	790.6
KD8	475.8	1092.4
KD9	3603.7	8294.5

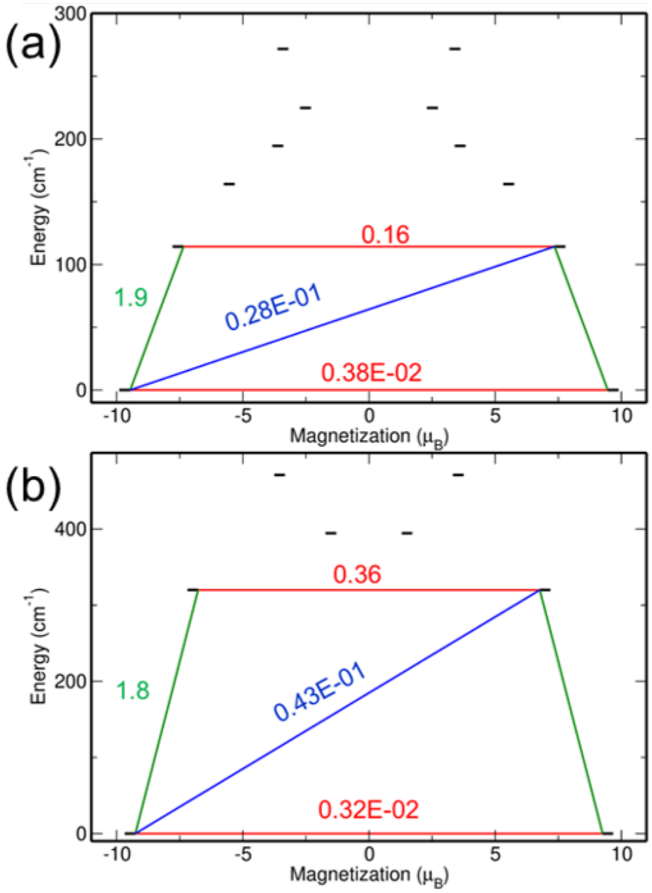

Figure S5: Comparison of blocking barrier of (a) $\mathbf{1}_{me}$ and (b) $\mathbf{2}_{me}$ computed using SA-CASSCF-SO level of theory and BS1 basis set combinations using SINGLE-ANISO module. The red lines indicate QTM or TA-QTM processes between $|\pm m_J\rangle$ states. The green and the blue lines indicate the transitions between the inter KDs (via Orbach and/or Raman mechanisms). The values correspond to transition magnetic moment matrix elements (in μ_B) between the m_J levels.

Table S8: Comparison of g-tensor values for $\mathbf{1}_{me}$ and $\mathbf{2}_{me}$ at the SA-CASSCF-SO level of theory using BS1 set of basis sets.

	$\mathbf{1}_{me}$			$\mathbf{2}_{me}$		
	g_x	g_y	g_z	g_x	g_y	g_z
KD1	0.01	0.01	19.35	0.00	0.01	18.93
KD2	0.42	0.52	15.93	0.82	1.26	14.71
KD3	1.43	1.85	14.24	1.49	2.46	14.87
KD4	1.39	3.91	8.88	8.58	4.80	0.54
KD5	3.14	5.81	9.75	3.23	4.51	9.93
KD6	0.21	0.40	18.39	0.22	0.35	17.81
KD7	0.03	0.06	19.03	0.03	0.04	18.27
KD8	0.00	0.01	19.69	0.01	0.01	19.06

Table S9: Comparison between the ab initio derived crystal-field parameters for the $\mathbf{1}_{me}$ complex and $\mathbf{2}_{me}$ complex computed at the SA-CASSCF-SO level with BS2 basis set.

k	q	B_k^q for $\mathbf{1}_{me}$	B_k^q for $\mathbf{2}_{me}$
2	-2	-6.49E-01	-1.04E+00
	-1	-1.39E+00	-3.42E+00
	0	-1.30E+00	-2.59E+00
	1	3.34E+00	6.33E+00
	2	7.59E-01	8.85E-01
4	-4	1.30E-02	2.67E-02
	-3	-1.71E-02	-4.52E-02
	-2	-7.77E-05	-9.87E-03
	-1	1.35E-02	3.01E-02
	0	-4.08E-03	-1.34E-02
	1	1.18E-03	-1.41E-03
	2	2.75E-02	6.71E-02
	3	5.63E-02	9.66E-02
	4	5.95E-03	2.35E-02
6	-6	-4.47E-05	-2.25E-04
	-5	4.42E-05	2.27E-04
	-4	-2.87E-05	-1.15E-04
	-3	8.97E-05	4.46E-05
	-2	-9.78E-05	-1.89E-04
	-1	-3.46E-05	-7.83E-05
	0	-5.27E-06	-7.47E-06
	1	-1.64E-04	-2.21E-04
	2	-5.28E-05	-1.27E-04
	3	2.44E-04	6.06E-04
	4	1.50E-04	2.39E-04
	5	1.77E-04	6.99E-04
	6	1.15E-04	1.74E-04

Table S10: Comparison between the spin-orbit energies (cm^{-1}) for $\mathbf{2}_{me}$ complex using different active spaces at the SA-CASSCF-SO level with BS2 basis set.

	SA-CASSCF(9,7)-SO	SA-CASSCF(9,12)-SO
KD1	0	0
KD2	329.0	434.9
KD3	398.9	706.8
KD4	481.0	1082.8
KD5	544.8	1551.6
KD6	664.2	2165.4
KD7	813.7	2905.9
KD8	1107.7	3857.6

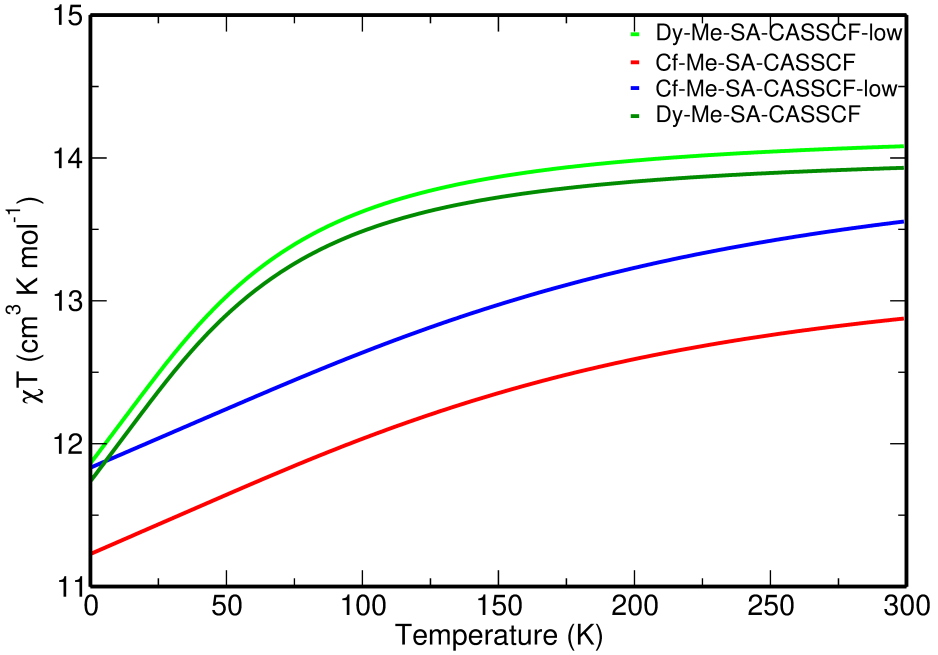

Figure S6: Comparison of the computed χT vs T curves of $\mathbf{1}_{me}$ (or Dy-Me) and $\mathbf{2}_{me}$ (or Cf-Me) complexes using SA-CASSCF-SO and SA-CASSCF-SO-low level of theory and the BS2 basis set.

Table S11: Relative energies (cm^{-1}) of the first 9 Kramers doublets of $\mathbf{1}_{me}$ and $\mathbf{2}_{me}$ using SA-CASSCF-SO and SA-CASSCF-SO-low level of theory (using BS2 basis set).

	$\mathbf{1}_{me}$		$\mathbf{2}_{me}$	
	SA-CASSCF-SO-low	SA-CASSCF-SO	SA-CASSCF-SO-low	SA-CASSCF-SO
KD1	0.0	0.0	0.0	0.0
KD2	120.1	118.3	363.1	329.0
KD3	171.3	169.6	406.3	398.9
KD4	201.9	199.9	516.6	481.0
KD5	233.7	232.0	581.5	544.8
KD6	283.1	278.3	741.3	664.2
KD7	363.0	356.7	911.2	813.7
KD8	499.2	490.8	1238.6	1107.7
KD9	3045.4	3599.4	5864.6	8280.9

Table S12: Comparison of relative energy (cm^{-1}) of 21 sextet roots of $\mathbf{1}_{me}$ and $\mathbf{2}_{me}$ complex using SA-CASSCF and XMS-CASPT2 level of theory (using BS2 basis set).

	$\mathbf{1}_{me}$		$\mathbf{2}_{me}$	
Root No.	SA-CASSCF	XMS-CASPT2	SA-CASSCF	XMS-CASPT2
1	0.0	0.0	0.0	0.0
2	5.7	7.6	32.5	73.7
3	153.1	210.6	373.8	476.9
4	177.8	231.1	454.3	543.0
5	208.2	301.7	667.2	808.4
6	309.5	413.1	840.9	1019.8
7	329.0	435.4	885.9	1013.5
8	383.1	488.9	964.0	1140.7
9	396.5	514.7	1015.2	1214.0
10	553.4	689.6	1413.0	1613.8
11	556.7	695.9	1437.8	1655.6
12	7606.0	6073.9	5659.6	4030.2
13	7634.8	6106.3	5820.1	4236.7
14	7759.5	6273.9	6106.3	4544.7
15	7776.3	6302.8	6213.2	4633.9
16	7792.8	6321.5	6304.1	4813.1
17	7839.3	6379.8	6366.7	4900.0
18	7864.6	6394.0	6510.3	4975.8
19	34904.1	27926.4	25177.8	18625.7
20	35142.2	28236.7	25709.2	19307.3
21	35315.9	28432.9	25981.2	19645.9

Table S13: Computed g-tensor values of $\mathbf{1}_{me}$ and $\mathbf{2}_{me}$ complex using SA-CASSCF-SO-low and XMS-CASPT2-SO level of theory (using BS2 basis set).

	$\mathbf{1}_{me}$						$\mathbf{2}_{me}$					
	SA-CASSCF-SO-low			XMS-CASPT2-SO			SA-CASSCF-SO-low			XMS-CASPT2-SO		
	g_x	g_y	g_z	g_x	g_y	g_z	g_x	g_y	g_z	g_x	g_y	g_z
KD1	0.01	0.01	19.48	0.01	0.01	19.48	0.00	0.00	19.45	0.00	0.00	19.47
KD2	0.43	0.54	15.99	0.32	0.43	15.88	0.84	2.25	14.02	0.75	1.79	14.54
KD3	1.39	1.89	14.43	1.65	2.72	14.27	0.49	2.03	15.53	0.70	1.86	16.05
KD4	1.69	4.21	8.68	1.61	4.72	8.24	2.04	4.86	9.49	2.15	5.15	9.18
KD5	3.01	5.55	10.08	2.81	5.38	10.62	2.74	4.73	11.65	2.56	4.64	11.79
KD6	0.21	0.38	18.54	0.18	0.30	18.67	0.11	0.19	18.54	0.10	0.22	18.45
KD7	0.02	0.06	19.15	0.03	0.05	19.18	0.04	0.09	18.95	0.03	0.09	18.98
KD8	0.00	0.01	19.78	0.00	0.01	19.78	0.02	0.04	19.58	0.02	0.04	19.59
